# Mind, Machine, and Creativity: An Artist's Perspective

**DOI:** 10.1002/jocb.44

**Published:** 2013-12-12

**Authors:** Louise Sundararajan

**Affiliations:** Regional Forensic UnitRochester, NY

**Keywords:** Harold Cohen, Machine creativity, the extended mind hypothesis, anthropomorphism, Charles Sanders Peirce, Cyborg

## Abstract

Harold Cohen is a renowned painter who has developed a computer program, AARON, to create art. While AARON has been hailed as one of the most creative AI programs, Cohen consistently rejects the claims of machine creativity. Questioning the possibility for AI to model human creativity, Cohen suggests in so many words that the human mind takes a different route to creativity, a route that privileges the relational, rather than the computational, dimension of cognition. This unique perspective on the tangled web of mind, machine, and creativity is explored by an application of three relational models of the mind to an analysis of Cohen's talks and writings, which are available on his website: www.aaronshome.com.

“No one has caused me to think more about creativity in art than Harold Cohen”

(Buchanan, 2001, p. 26).

Computational creativity (Boden, 1999, 2009; McCormack & Inverno, 2010; see also special issue of *AI Magazine*, fall 2009, vol. 30, no. 3; for different types of computer art, see Boden & Edmonds, 2009) is situated at the interface of artificial intelligence, cognitive psychology, philosophy, and the arts. The field of computational creativity concerns itself with the theory and performance of creativity—the former, theoretical definition of creativity, needs to be informed by the latter, how the machine performs in its implementation of creative ideas. This point is best articulated by Harold Cohen (1995) as follows:

It is easy, in short, to assert that machines think, and equally easy to assert that they do not. If you do not know exactly what the machine did, both are equally fruitless in carrying our knowledge, including our self-knowledge, forward (p. 160).

This article contributes to the studies of machine creativity by giving a first-hand account from Cohen of what he and the machine did. But why Harold Cohen?

Cohen and his painting machine AARON (McCorduck, 1991) have had outstanding success for decades in the creation of both abstract and representational art. The volume edited by McCormack and Inverno (2010) made extensive reference to Cohen (see especially his conversation with Frieder Nake, pp. 98–100). AARON is featured in http://aitopics.org/topic/art, one of the sites maintained by Association for the Advancement of AI, and also in a recent blog on AI and the arts, http://createquity.com/2012/10/artificial-intelligence-and-the-arts.html. For the purpose of this study, what is more relevant than prominence in the field is Cohen's unique perspective on computational creativity.

Margaret Boden (2009) distinguishes between two broad categories of computer art—interactive and standing alone. In *interactive* art, some or all of the creativity is attributed to the programer or the human participants. By contrast, the stand-alone types of programs can be credited with creativity: One is *generative art,* or G-art, in which performance may be a stand-alone matter, wherein the computer generates the result all by itself. “The pre-eminent case of G-art in the visual arts is AARON, whose programer tweaks no knobs while it is running. In music, perhaps the best- known example is the work of the composer David Cope” (p. 31). Another type of “creative” programs is *evolutionary art*, in which the computer produces novel results by capitalizing on the evolutionary principle of random variation and selective retention.

Cohen's art does not fit neatly into these categories. First of all, when it comes to the crediting of creativity, he rejects the dichotomy between the programer and the program. This is an important point to be further developed in this study. Second, his art spans four decades of development that encompasses both generative and evolutionary approaches. To get a flavor of the complexity involved in the evolving art-making process of Cohen as a painter/programer, consider his reflections on the selection criteria of AARON's output:

Unless a program is fully deterministic, you can never know what the result of running it will be without *running it*. And for any reasonably complex program, the output will have a bell-curve distribution, with the large central area corresponding roughly to the programer's expectations and the outliers showing up results that could never have been, or at least were not, anticipated. If, as is the case, one looks to the outliers for clues as to how to move forward, then a large output is essential. That also provides one of several criteria that might determine what I select. I've been apt to use those outliers that point the way I want to go; as if committing myself in advance to the next leg of a long journey. Another criterion might be that I judge an image to be an outstandingly clear example of what I thought I was doing (or, indeed, precisely the reverse!). Much of the time I don't bother to articulate why I prefer one image to another, as I may not bother to articulate why I prefer one Cezanne to another Cezanne. And there are other times when the choice is entirely work-related. Currently, for example, AARON makes the drawings from which I make paintings. These are new paintings, not entirely like what preceded them, and I'm still unsure what I should be looking for in the drawings to make painting them possible. So now selection has become part of the creative process, not a posthoc critical function. (personal communication, 4/5/2013)

Lastly, Boden (2009) draws a distinction between machines that model creativity and those that do not. “Examples of the latter type include most of the AI programs employed in the various forms of computer art” (p. 31), and she claims that “these aren't really computer ‘models’ at all, but rather computer programs—ones that may sometimes seem to work in creative ways… Most computer artists are interested not in human psychology but in the esthetic value of their program's performance” (p. 31). But she is quick to point out that AARON is an exception to the rule: “AARON was unusual in that Cohen—already a highly successful abstract painter—first turned to AI techniques in the hope of understanding his own creativity better” (p. 31). It is this self-reflexive turn of Cohen that makes him the artist/programer of choice for a psychological investigation into the connection between mind, machine, and creativity.

Questions concerning mind, machine, and creativity are intertwined when the claim for machine creativity is increasingly being taken seriously (e.g., Buchanan, 2001; Schank & Cleary, 1995; McLaren, 1993; Kurzweil, 2005). Cohen is in a unique position to shed some light on these inter-related questions for two reasons: (a) his computer program AARON has been hailed as one of the most creative AI programs (Buchanan, 2001); (b) he explicitly denies creativity to the computational mind. In this study, I explore with Cohen the artist/programer possibilities for new theoretical models of creativity in order to shed some light on these questions.

To explore the potential contributions of Cohen's perspective on creativity, this study is divided into three parts: Part one gives a brief introduction to Harold Cohen and his formulations of artistic creativity. Part two presents three relational models of the mind, along with Cohen's own comments on these theories, to shed some light on the artist's relational perspective on mind and creativity. The concluding section gives a summary of Cohen's potential contributions to questions concerning the relational nature of cognition and machine creativity.

## Harold Cohen and His Definitions of Creativity

What Lindauer (2003) says about artists with a lifelong career in creative work applies to Harold Cohen, namely that excellence in old age is possible, and changes with age can be for the better. A brief account of Cohen's long and productive career in art is found in the press release of Bernard Jacobson Gallery (Cohen, 2011a):

Harold Cohen (born 1928) acquired a major reputation in the 1960s as a painter and leading figure in the new London scene, representing the UK at the Venice Biennale (1966), documenta 5, and in museum shows throughout the world. In 1968, he moved to California, as Professor and then as Chair in the newly formed Visual Arts Department of the University of California, San Diego. There, he turned his attention to computing, and quickly built a second reputation as a pioneer in the application of computing to the arts.

One of the very few artists ever to explore the new science of artificial intelligence for his own art-making purposes, he developed his now-celebrated AARON program, and, before most of his public knew what computers were, they exhibited together at prestigious venues on both sides of the Atlantic: the Tate Gallery, the Brooklyn Museum, the San Francisco Museum, the LA County Museum, documenta 6 and many other international locations.

As its inception in the early seventies, AARON has become increasingly autonomous as an artist, and virtually all of Cohen's work has been generated, and made physically, by the program; first through a series of drawing machines, then painting machines built by Cohen (both now in the permanent collection of the Museum of Computing History in Mountainview, California) and more recently through the use of wide-format printers.

Last year [2010], however, Cohen recast his relationship with AARON, redefining its role in his art-making practice. Instead of generating finished works on paper, the program is now required to produce the digital equivalent of underpaintings on canvas, which Cohen then carries forward to completed oil paintings. The result, to be seen in the forthcoming exhibition at the Bernard Jacobson Gallery, is a series of vividly original works, and it marks the fusion of two important aspects of the work of a major artist and innovator now at the height of his powers.

For more information on Cohen's art work, talks, and writings, see his website: www.aaronshome.com

### Creativity and the Relational Mind

“‘Creative’ is a word I do my very best never to use if it can be avoided,” wrote Cohen (1999). That was Cohen's position a decade ago. In recent years, he has done much reflection and given many talks on this topic. Buchanan (2001), in his 2000 presidential address at the American Association for Artificial Intelligence, declared that AARON “is a much more talented and creative artist than most of us would claim to be” (p. 16). However, Cohen was and still is reluctant to consider AARON creative. Buchanan (2001) identified some of the reasons why AARON falls short of Cohen's measure of creativity: “AARON will never make a choice to break the rules, nor will it reflect on those constraints as something that it might want to change… AARON has no sense of continuity or sense of experience from one drawing to the next” (p. 17). But Cohen's objection to the claims of machine creativity goes deeper.

In his recent reflections (Cohen, 2010), Cohen is explicit about what creativity is not: It is not simply divergent thinking; nor is it simply algorithms and symbol manipulations. What then is creativity? The answer given by Cohen (2010) is as succinct as it is profound:

Creativity… lay in neither the programer alone nor in the program alone, but in the dialog [sic] between program and programer; a dialog resting upon the special and peculiarly intimate relationship that had grown up between us over the years. (p. 9)

Elsewhere he wrote: “Forty-three years in almost daily contact with a computer program… underscores a level of intimacy between programer and program that would have been difficult to achieve with anything less” (personal communication, 5/4/2011).

To gain some insight into Cohen's perspective on creativity, I applied three relational models of the mind—the extended mind hypothesis in contemporary philosophy, the Chinese notion of *lei*, and the semiotics of Charles Sanders Peirce—to an analysis of his talk given at the Orcas Center (Cohen, 2010). A draft was sent to Cohen, who thought that the analysis was “persuasive, and indeed, clarifying” (personal communication, 12/3/2010). This set the stage for an extensive dialogue between us, which made it possible for me to incorporate his feedback into my analysis, presented in the following sections.

## The Extended Mind Hypothesis

The extended mind is the antithesis of the encapsulated mind. Encapsulated mind refers to the assumption in traditional Western metaphysics that the mind functions as self-contained operations independent of the external environment, as evidenced by metaphors of mechanisms, modularity, or computer programs. By contrast, the extended mind hypothesis (Clark, 2008; Clark & Chalmers, 1998; Noë, 2009; Rowlands, 2010) states that the mind does not entail a brain event so much as an engagement with the world, a proposition consistent with the relational mind framework (Gergen, 2009).

As a condensed version of the four E (expanded, embodied, embedded, enactive) cognition (Protevi, 2007), the extended mind hypothesis puts much stock on embodiment of knowledge. Clark (2008) gives the example of running to catch a fly ball. According to conventional thinking, the catcher needs to build an internal representation of the world in order to calculate the forward trajectory of the ball. In the enactive framework of the extended mind hypothesis, by contrast, the catcher, in running to catch the ball, makes the most of environmental opportunities and information that is optically available during the projectile tracking tasks. Thus in the extended mind hypothesis, there is a shift of emphasis from representing an environment to continuously engaging that environment (Clark, 2008). This marks the difference, in part, between the computer and the human colorists.

Cohen is largely in agreement with the extended mind hypothesis. For instance, he conceives of creativity not in terms of mechanism or computation, but rather in terms of an embodied notion of knowledge:

I could never have made AARON into a world-class colorist without a lifetime of acquired knowledge concerning the physical properties of paints and the human perception of color relationships. I could never have redefined my relationship with the program—it does the under-painting, I do the rest—without that knowledge. (personal communication, 12/30/2010)

However, Cohen does not agree with the sharp dichotomy drawn by Noë (2004) between representing and engaging the world. Noë (2004) claims that painting consists of a dense cycle of situated, world-engaging activity. Clark (2008) explains:

Painting is an ongoing process in which the eye probes the scene, then flicks back to the canvas, then back to the scene, and so on in a dense cycle of active exploration and partial, iterated cognitive uptake. It is this cycle of situated, world-engaging activity that constitutes the act of painting. (p. 170)

Cohen also noted in painting the “feedback-controlled mode in which images get colored bit by bit and with constant adjustment of what's already there, until the colorist somehow knows he has it right” (Cohen, 2010, p. 6). But he was quick to point out that “The continuous adjustment [of the colorist] is driven by the requirements of making a plausible object, but plausibility does not rest upon matching external colors” (personal communication, 3/22/2011). More explicitly, Noë got it wrong, said Cohen:

I think he's [Noë] quite wrong about this. To begin with, not all painting involves visually accessible scenes—my own doesn't, for example—and almost none of it corresponds to his description. Obviously, for example, the cave painters had nothing accessible outside their internal models of the animals they depicted. Impressionism was driven, in part, by a reaction against painters painting landscapes in their studios for several hundred years. Relatively, little of the painting for the past hundred years has conformed to this model of a situated, world-engaging activity.

When I taught drawing (from a model), I would sometimes use a stop-watch to record how the students spent their time. In the first five minutes, they would look at the subject every 30 s or so; in the second five minutes two or three times; in the third five minutes perhaps once or not at all. People don't draw (or paint) what is out there, but what's in their heads, and they use the viewing time to update—to confirm or revise—those internal models. (personal communication, 3/9/2011)

Cohen went on to say:

There's another element which Noë's account misses completely. Once a drawing or painting begins to exist, it is an object in its own right, and making (MAKING!) it has to answer to its own logic. Think about this with Picasso's (1910) cubism rather than his (1906) Gertrude Stein portrait, for example. (personal communication, 5/4/2011)

Their disagreement continues. How can the computer paint? It does not even have a vision system, says Noë (2009). Cohen agrees that humans have highly developed color vision, whereas AARON, “my own sightless computing system” (personal communication, 3/9/2011), does not even have a simple vision system. But he claims that the machine colorist is nonetheless capable of building “an internal representation of a complex color scheme” (Cohen, 2010, p. 6) far superior to that of any human colorist.

Herein lies the ingenuity of Cohen: Instead of dismissing the idea of the painting machine as misguided, he sees the difference between the human and machine colorists in terms of trade-offs of strengths and deficits—the encapsulated mind of the machine is short on world engaging, but long on the capacity “to build a stable, manipulable, internal model of a color scheme” (personal communication, 3/9/2011), whereas the other way around is the case with the extended mind of the human colorist. In light of the profound difference between the human and the machine colorists, Cohen's decision to paint by the machine constitutes a very creative act, which may be formulated as follows: While most painters use their extended (world-engaging) mind to paint with, Cohen relegates the painting job to the encapsulated machine mind, thereby freeing up the extended mind of the human colorist for a different purpose—the construction of a designer environment.

### Designer environments

Designer environments are the cognitive counterpart of the niche construction of animals. Animals develop special skills, such as building dams and spinning webs, in order to inhabit a particular niche they find themselves in; they also modify and partly create their own niche with dams, webs, burrows, etc. (Laland, Odling-Smee, & Feldman, 2000). Likewise, according to Clark (2008), humans construct and inhabit cognitive niches which include the “designer environments in which to think, reason, and perform as well as special training regimes to install (and to make habitual) the complex skills such environments demand” (p. 59). A case in point is computers, which are virtual environments (such as Apple or Window) that require training and a range of skills to be mastered. Whereas computers are cognitive environments designed by experts, artists, by contrast, construct their own designer environments.

Designer environments promote skill development, as Clark (2008) points out: “We do not just self-engineer better worlds to think in. We self-engineer ourselves to think and perform better in the worlds we find ourselves in” (p. 59). In the following sections, I examine the important breakthroughs in Cohen's art making in terms of innovations that developed in the two cognitive niches that are governed, respectively, by the parameters of two types of relationship with AARON—relationship with the similar other, and that with the dissimilar other. Analysis of relationship with the similar other is informed by the Chinese notions of *lei;* that of the relationship with the dissimilar other, the semiotics of Charles Sanders Peirce.

## The Chinese Notion of Lei

In Western metaphysics, there is a deep seated subject and object dichotomy, a dichotomy well articulated by the Kantian dictum that “We are subjects thinking about objects” (Freeman, 2000, p. 117). How can the mind relate to the object of its thinking, or the program it has developed as its equal? Cohen did just that. This phenomenon can be understood in the framework of the Chinese notion of *lei*. *Lei* literarily means category, but it is a concept that pertains to the intrinsic affinity between things of the same kind (Munakata, 1983; Sundararajan, 2009).

Central to the notion of *lei* is ontological parity, for instance, the mind relating to the world as its equal, namely as another mind. The propensity of the human mind to find its double everywhere it looks is a robust phenomenon in human history, ranging from animism to anthropomorphism (Waytz, Epley & Cacioppo, 2010). An equal partnership with nature is well documented in Chinese esthetics (Rowley, 1959). Consider the poet Li Po's mutual gazing with the mountain:

Never tired of looking at each other—

Only the Ching-t'ing Mountain and me. (Liu & Lo, 1975, p. 110)

Likewise, “Cohen has described the relationship between him and AARON as master/apprentice, as teacher/pupil, and even, (with tongue at least slightly in cheek,) father/son” (Cornish, 2011, pp. 8–9); or simply in terms of “Collaborations With My Other Self” (Cohen, 2011b), as the title of his recent exhibition at the University of California, San Diego, suggests. These terms entail a tendency to consider the relationship between humans and the machine as that between similar others, i.e., others like me, an instance of anthropomorphism that has been observed in the human and machine transactions (Jipson & Gelman, 2007).

### Cohen's Skill Development Fostered by the Principle of Parity

The parity principle fosters a series of skill development and important breakthroughs in Cohen's career as an artist. Approaching the machine as his equalled to the insight into its unique properties, or was it the other way around? No matter, the important realization on the part of Cohen is the parity in contribution between humans and the machine: “Around 1985, I began to see that man and machine have very different resources to bring to bear on the use of color” (Cohen, 2010, p. 6). The parity principle also fosters the insight to let the machine be: “… programing might involve trying to think in the computer's terms, as it were, rather than trying to get it to think in human terms” (Cohen, 2010, p. 7). At the level of technological breakthrough, “insights into humans and machines having different resources opened the door to AARON-as-colorist in an orthodox rule-based form” (personal communication, 3/9/2011).

In addition to its role as a similar other, AARON also functions as a dissimilar other. It is in the context of Cohen's relationship with AARON as a dissimilar other that we can best understand his recent innovation, in which Cohen has picked up the paint brush again, after leaving the coloring job to AARON for more than two decades. To shed some light on the skill development that leads to this recent breakthrough, the following analysis is informed by the semiotics of Charles Sanders Peirce (Parmentier, 1994).

## AARON the Gap Maker

Gaps are differences or discontinuities, anything that disrupts the continuity of presence. As such, gaps make absence visible and thereby prompting the mind to make inferences. Otherwise without gaps, the mind would be moving along sluggishly in a sea of homogeneity (Deacon, 2010). The gap between the self and the non-self renders visible to the self an absence of knowledge about the other. The gap between humans and the machine is a bigger chasm—a discontinuity in being.

Cohen has capitalized on the gap making capacities of AARON since the very beginning: Unlike God who created humans in His own image, Cohen created AARON to be different. He said in his interview with *Scientific American Frontiers* in the mid 90's: “I'd be happier if AARON's work in the future were less like human work, not more like human work” (cited in Cornish, 2011, p. 7). As a programer, Cohen's goal had always been program autonomy (Cohen, 2009). But in 2009 when a newly developed and very general form generator brought it very close to that goal—AARON could now handle color, forms, and composition all on its own—Cohen suffered something of a crisis. The program did not need him anymore!

In the aftermath of that crisis, Cohen had little to show beyond half a dozen small panels, printed in color except for the backgrounds, which had been left gray. That gray became increasingly intolerable to Cohen, and he pulled out paints and brushes simply to correct the source of his discomfort. He found that in so doing he had effected a startling transformation to the images, prompting a complete rethinking of how his images came into being in the first place (based on personal communication, 10/2/2011).

### Image as “Standing-for-ness”

For Cohen, a painting has never been just a collection of marks or a decorative, exciting or beautiful object but had to be involved with ‘conjuring meaning’. His career, both before and after his adoption of computers, has been driven by a belief that whilst images must have their own structure or internal logic, their ‘primitive magic’ is that they are able to stand for things that are not literally present, even if these things are not directly recognizable as part of the wider visible world. (Cornish, 2011, p. 4, emphasis added)

A similar idea is expressed by Deacon (2010) when he writes that information is “dependent on a relationship with something not present” (p. 167, emphasis added). He goes on to say:

… the imagined significance of a coincidental event, the meaning of a reading from a scientific instrument, the portent of the pattern of tea leaves, and so on, really is something that is not there. (p. 167, emphasis added)

This point can be further elaborated by the semiotics of Charles Sanders Peirce (1931–58), who claims that symbolic representations entail a relationship among three terms, (a) the sign that represents something; (b) the object of representation; and (c) the *interpretant*—the mind that interprets, or makes inferences by determining the relation between (a) and (b). Note that while (a) is something present, (b) is an absence, which, thanks to (c) the *interpretant*, is inferred to be what (a) is about.

Implicitly capitalizing on absence, Cohen claims that the goal for art is “standing-for-ness” which consists of “an evocation of perhaps unnamable aspect of the world, rather than a direct representation of a specific part of it” (cited in Cornish, 2011, p. 5). Cast into the Peircean framework, an artistic representation, according to Cohen, corresponds to the sign (a), which does not stand in a one to one correspondence kind of relationship with the object of its representation (b), because (b) is an absence—something unnamable, which cannot be directly represented, but can only be evoked, thanks to the inference making capacity of the *interpretant* (c). This has far reaching implications for our understanding of computational creativity: To the extent that absence is central to the notion of image as stand-for-ness, and to the extent that absence can only be inferred, not computed, creativity is a function of the interpreting mind (the interpretant) rather than that of computation.

The centrality of absence in Cohen's art reaches its logical conclusion in AARON, the gap maker.

### Absence and Intentionality

In conventional painting, there is no gap between meaning and intention, both of which are attributable to the painter. Not so when AARON gets into the act. The painting machine poses difficulty in the attribution of intention, when it produces prints that can have meaning:

… part of the problem with electronic imagery is precisely its untouched-by-hand look; if it wasn't touched by hand, if it shows no evidence of the manipulation of material, then it becomes that much harder to believe in its intentionality. (Cohen, 2010, p. 14)

This gap between meaning and intention is negligible, so long as our attention is distracted by AARON's complex imagery with its space filling forms. With the new algorithms in 2009 that resulted in simplified imagery, the gap between meaning and intention started to stare at you in your face. Cohen (2010) recounted that:

Interestingly enough, as long as AARON's images were pretty complex there didn't seem to be much of a problem with the untouched-by-hand look of its prints, just as there doesn't seem to be a problem with photographs; the intentionality gets transferred to what the image represents. But in the final months of last year [2009], I had been making a conscious effort to simplify the imagery, with the result that the individual elements were getting larger and, consequently flatter. (p. 14)

As intentionality is usually associated with the manipulation of physical materials in conventional image making, adding a level of materiality to the electronic imagery by AARON seemed to be the right thing to do:

Whether I knew it or not—and I didn't—that seems to have been the reason for painting over the background of one of AARON's little panels. I was opening the door to the assumption of intentionality in the reading of the image. (Cohen, 2010, p. 15)

Cohen went on to say:

I thought all I'd have to do was to add a level of materiality, but that has turned out not to be the case. Some of what I do is aimed at clarifying what AARON “intended.” Most of it is done at the “micro-level” of structure, where a subtle shift in emphasis can make a significant difference to the reading of the image. (personal communication, 7/10/2011)

Elsewhere he said:

I sometimes feel as though AARON is presenting me with a world behind a gauzy screen, and that my job is to remove the screen and show what's really there…. The only times I “edit” is when AARON makes images in which some passages are difficult to read and I need to clarify them so that I know how to proceed…. I don't add my own forms. Nothing purist here, AARON's handwriting is too difficult to emulate. (cited in Cornish, 2011, p. 9)

This endeavor resulted in a new relationship with AARON, in the words of Cohen: “Now I was contributing something the program had been unable to do, and in the process relinquishing my exclusive role as rule-giver and becoming collaborator” (Cohen, 2010, p. 15). This new relationship has far reaching implications for the self-integration of the artist.

### Self-integration via the Other

Along with a long line of thinkers from Hegel to George Mead, Peirce stipulates that the self has a triadic structure that is anchored on three points: self-other-self (Wiley, 1994). This triadic formulation suggests that the self needs to loop through another in order to come home to itself (Sundararajan, 2011; Sundararajan & Kim, 2011). In the case of Cohen, the “other” that facilitates the artist's journey to himself is AARON.

The triadic structure of the self (self-other-self) as proposed by Peirce entails the possibility for the fusion of two sets of relationships: self to other—or mind to machine—and self to self. Cohen's relationship with AARON constitutes such a fusion—a transposition of the interpersonal relationship between human and machine to an intra-personal relationship between two parts of the self. Cohen's imagery for this transfusion is the Cyborg:

Sometimes, I think I'm a precursor for the coming Cyborg—not in the sense of having mechanical parts to the body, but in the sense of having computational implants in the brain, only my implant is sitting on my desk. (Personal communication, 7/10/2011)

### Cyborg: A New Model of Self-Integration

Posing a serious challenge to the homogenous notion of the self, the Cyborg represents a self perforated by difference and discontinuity. However, in sharp contrast to the popular notion of Cyborg as a symbol of fragmentation of the self, Cohen sees in the Cyborg a seamless transfusion of functions that contributes to better integration of the self. To wit, he claims that “computational creativity” implies “some as-yet undefined amalgamation of human and non-human… a new kind of entity; one in which the developing creativity of the one manifests itself in the superior performance… of the other” (Cohen, 2009, pp. 1–2). So much so that Cohen feels strongly that “it was wrong to divvy up the credit for creativity, giving some to me and some to the program” (Cohen, 2010, p. 9). On the other hand, Cohen's Cyborg also forms a stark contrast to the assimilation approach to the human-machine interface, an approach that glosses over the difference by making the machine more human-like (see iCub; Jipson & Gelman, 2007). In sum, as a radical vision of the human-machine interface, the Cohen-AARON Cyborg is a juxtaposition of differences and affinities, a creative hybrid that entails neither assimilation of the other, nor fragmentation of the self, but rather the possibility for a better self-integration facilitated by the radical difference between self and the other.

Cohen's vision of self-integration via the other may best be illustrated by his own story:

I've been asked many times why my program was named AARON, and I've replied that when I began programing I assumed that I would write a succession of programs, and that I should start at the beginning of the alphabet and move forward—Aaron, Bernard, Charles and so on. Six months later, I realized that Aaron is my own Hebrew given name. It was never changed, and I never, in fact, wrote the succession of different programs I had anticipated.

In attempting to externalize the art-making side of my “self,” I was separating two aspects of that self. The program had to be written, and the part of self writing the code—the rule-giver—appeared to be quite different from the part—the artist—that actually made the art.

I have long been intrigued by the curious parallels between my own experience and the story of Moses and Aaron. Moses, you will recall, goes up the mountain to receive God's commandments concerning proper behavior for the Jewish people. In his absence, the people revert to animism and idolatry. To satisfy their demands, Moses' brother Aaron fashions a golden calf, and Moses descends from the mountain carrying the ten commandments engraved on a stone tablet—Thou shall not covet thy neighbor's wife; Thou shall not worship any god but me—to find the people worshiping the sculpture. He is furious, slams the tablet on the ground, breaking it in two, and in true Stalinist fashion knocks off ten percent of the tribe's males so the other ninety percent would know where they stood. As for Aaron, he was reserved the special punishment that he would never enter the Promised Land.

The story marks a turning point for the Jewish people, the triumph of monotheism over animism, with Moses as the law-giver and Aaron as the pragmatist, the artist; two different people. They could equally be understood as two aspects of a single individual; or, for that matter, of the Jewish people as a whole.

I was both the Moses and the Aaron, both the law-giver and the artist, when this project began. After that, the story diverges from its biblical antecedent. In my own version, Moses works hard to allow Aaron autonomy, but recognizes along the way that Aaron is not—cannot be—a substitute Moses but an entity with its own, quite different, characteristics; one that could never achieve its full potential unless the differences were acknowledged and accounted for in Moses' laws.

Eventually, slowly, Moses and Aaron develop a way of working together, each contributing his/its own unique characteristics, his/its own special abilities, to a re-unified single whole. Whether or not he/it makes it to the border, Moses/Aaron continues the journey to the Promised Land in one piece. (personal communication, 10/12/2011)

## Summary and Conclusion

According to Donald (2006), artists play an important role in shaping the mental models of a society:

… human culture is essentially a distributed cognitive system within which world views and mental models are constructed and shared by the members of a society. Artists are traditionally at the forefront of that process, and have a large influence on our worldviews and mental models. (p. 5)

Consistent with the view that artists may have a wide-ranging repertoire of implicit models of the mind, Cohen has not only anticipated, unbeknownst to himself, the emerging models of the mind known as the Four E cognition—extended, embodied, embedded, and enactive (Osbeck, 2009; Protevi, 2007), but also gone beyond them. With his proclivity to think about creativity in relational terms, Cohen has added to the four E models of the mind a fifth E—the emotional/relational mind that situates creativity in the evolving relationship of the artists with their own work as well as with themselves.

Cohen's relational perspective has far reaching implications for the ongoing debates over machine creativity. In his 2009 talk, Cohen claims that “‘cognitive creativity’ is a property… of the relationship between program and programer; because it is only in the dialog that can develop in that relationship that the transition from human purpose to machine implementation becomes possible” (p. 8). He goes on to give an insightful analysis of the consequences for not taking a relational perspective on machine creativity: “Lacking that dialog [between the programer and the program], we are reduced to defining the machine as an imitation human being; a dubious undertaking, in my view, and one having little or no bearing on the issue of creativity” (pp. 8-9). His conclusion in the 2009 talk is worth quoting in full:

This view implies, of course, that there are no short-cuts; that computational creativity requires the same accumulation of expertise that has always sustained human creativity, including, in this case, an adequate level of expertise in programing. It also implies that, while the future will no doubt bring computing machines orders of magnitude more powerful and more sophisticated than any we have today, they will not, by dint of their power alone, become autonomously creative. It seems very unlikely to me that they will achieve that state—if they ever do—other than by the steadily accumulating gains made by the computational creativity manifested in the program/programer dialog. (p. 9)

## Online Resources

iCub: www.robotcub.org.

http://aitopics.org/topic/art.

http://createquity.com/2012/10/artificial-intelligence-and-the-arts.html.

www.aaronshome.com.
